# EVERYDAY DIGITAL LITERACY QUESTIONNAIRE (EDLQ): DEVELOPMENT AND VALIDATION FOR KOREAN OLDER ADULTS

**DOI:** 10.1093/geroni/igad104.2204

**Published:** 2023-12-21

**Authors:** Jiyoung Shin, Jiyeon Choi, Seongmi Choi, Kijun Song, Heejung Kim, Mona Choi, Yesol Kim

**Affiliations:** Yonsei University, Seoul, Republic of Korea; Yonsei University, Seoul, Republic of Korea; Yonsei University, Seoul, Republic of Korea; Yonsei University, Seoul, Republic of Korea; Yonsei University, Seoul, Republic of Korea; Yonsei University, Seoul, Republic of Korea; Yonsei University, Seoul, Republic of Korea

## Abstract

Aging in the digitalizing society demands digital literacy, a core competency that involves identifying, evaluating, and communicating information using various digital devices or relevant programs. This study aimed to develop the Everyday Digital Literacy Questionnaire [EDLQ], a scale to assess digital literacy, and to evaluate its psychometric properties among community-dwelling older adults in South Korea. The EDLQ was developed using an instrument development design. A nationwide survey was conducted in 1,016 community-dwelling older adults aged 60 to 94 years. To evaluate psychometric properties, following analyses were conducted in two randomly selected subsamples (n=508 per each): internal consistency (Cronbach’s alpha), construct validity (exploratory factor analysis [EFA] and confirmatory factor analysis [CFA]), and concurrent validity with the eHealth Literacy Scale (e-HEALS). Among initial 30 items of the EDLQ, 22 items with a 3-factor solution showed 77% of explained variance in total. The subscales includes “Information and Communication” (9 items), “Contents Creation and Management” (4 items) and “Safety and Security” (9 items). CFA was conducted with this 3-factor solution (χ²/df=1.675, CFI=0.997, TLI=0.997, RMSEA=0.036, SRMR=0.050, CR=0.903-0.959, AVE=0.699−0.724, r²=0.616−0.773). Concurrent validity with the e-HEALS demonstrated a strong correlation (r=0.75). The Cronbach’s α coefficient was .98. Our study findings suggest psychometric properties of the 22-item EDLQ is valid and reliable to assess digital literacy for everyday technology use in Korean older adults. The EDLQ will be useful while developing technology-based interventions for community-dwelling older adults.

